# Prefix Stripping Re-Re-Revisited: MEG Investigations of Morphological Decomposition and Recomposition

**DOI:** 10.3389/fpsyg.2019.01964

**Published:** 2019-09-06

**Authors:** Linnaea Stockall, Christina Manouilidou, Laura Gwilliams, Kyriaki Neophytou, Alec Marantz

**Affiliations:** ^1^Department of Linguistics, Queen Mary University of London, London, United Kingdom; ^2^Department of Comparative and General Linguistics, University of Ljubljana, Ljubljana, Slovenia; ^3^Department of Psychology, New York University, New York, NY, United States; ^4^New York University Abu Dhabi Institute, New York University, Abu Dhabi, United Arab Emirates; ^5^Department of Cognitive Science, Johns Hopkins University, Baltimore, MD, United States; ^6^Department of Linguistics, New York University, New York, NY, United States

**Keywords:** morphological processing, lexical access, morphological recomposition, morphological decomposition, magnetoencephalography, derivational morphology, prefixation, grammatical licensing

## Abstract

We revisit a long-standing question in the psycholinguistic and neurolinguistic literature on comprehending morphologically complex words: are prefixes and suffixes processed using the same cognitive mechanisms? Recent work using Magnetoencephalography (MEG) to uncover the dynamic temporal and spatial responses evoked by visually presented complex suffixed single words provide us with a comprehensive picture of morphological processing in the brain, from early, form-based decomposition, through lexical access, grammatically constrained recomposition, and semantic interpretation. In the present study, we find that MEG responses to prefixed words reveal interesting early differences in the lateralization of the form-based decomposition response compared to the effects reported in the literature for suffixed words, but a very similar post-decomposition profile. These results not only address a question stretching back to the earliest days of modern psycholinguistics, but also add critical support and nuance to our much newer emerging understanding of spatial organization and temporal dynamics of morphological processing in the human brain.

## Introduction

Taft and Forster’s (1975) paper “Lexical storage and retrieval of prefixed words” is generally taken as the first clear model of complex word recognition in which initial decomposition of a complex word into its constituent morphemes is followed by the activation of the stored lexical entries of each of these constituents. Critically, Taft and Forster’s model [and its subsequent restatements in Taft (1981, 1988, 2004)] rests on the assumption that all affixes are detected and stripped by the same early, form-based, morphological parsing mechanisms. The model makes no distinction between prefixes and suffixes, inflectional and derivational affixes, or between bound and free stems. In fact, the 1975 paper starts with a review of evidence from suffixation experiments that complex words are parsed into their constituent stems and affixes (Gibson and Guinet, 1971; Snodgrass and Jarvella, 1972). It then goes on to present data from a series of experiments involving the nonword stems of prefixed words like *rejuvenate* and *resume*, which they take as evidence that prefix stripping is the first, obligatory, stage of lexical processing. The focus on prefixation in the 1975 paper, and in Taft (1981), was so influential on subsequent research on processing morphological complexity that Marslen-Wilson et al. (1994) writing in 1994, could state: “Under the influence of Taft and Forster’s (1975) affix-stripping hypothesis, a high proportion of the research in English has been on the perception of derivationally prefixed words.” (fn 3, p. 6).

However, in large part due to the research inspired by the results and methods of Marslen-Wilson et al. (1994) (see the section “But What About Prefixes:” for details), the majority of research on morphological processing in a wide range of languages over the intervening quarter century has focused on suffixed, not prefixed, words. This is particularly true of the research using neuroimaging tools to investigate the spatial organization, and temporal dynamics, of morphological processing in the human brain. A growing body of recent work using MEG provides a comprehensive overview of the timing and spatial distribution of a number of distinct steps in complex word processing, but all extant studies investigate suffixation. We build on this work to revisit the long-standing question about whether prefixed and suffixed words are processed by the same mechanisms.

Models of word recognition have long made a distinction between different stages of processing morphologically complex words. Taft and Forster (1975)’s explicit multi-stage processing model for visual word processing is, crucially, a serial model in which initial decomposition of a complex word into its constituent morphemes is followed by the activation of the stored lexical entries of each of these constituents (see also Taft, 1979, 2004). Only after these constituents have been activated, are the separate morphemes recombined into a complex whole word, which can then be interpreted. Following a tradition of modeling which assumes a pre- and a post-decomposition stage, Schreuder and Baayen (1995) propose what they call a meta-model of morphological processing, designed to account for both visual and auditory processing of complex words. The innovation of the model is that it proposes distinct post-decomposition stages, taking into account linguistic notions such as subcategorization. The three post-decomposition processes distinguished by Schreuder and Baayen (1995) are *lexeme lookup*, in which the constituent elements are retrieved from the mental lexicon, and their lexical features are activated, *licensing*, in which the consistent elements that have been detected are evaluated for whether they can be integrated based on their subcategorization properties (e.g., -able attaches to verbal stems), and *combination*, in which the lexical representation (syntax and semantics) of the whole word is computed on the basis of the lexical representations (syntactic and semantic) of the constituents which have been integrated.

Multi-stage processing models for morphologically complex words and pseudowords are robustly supported by a wide range of behavioral experimental and modeling data, but the availability of confirming neural evidence varies widely depending on the processing stage. There is now a sizeable body of converging evidence from behavioral, Electroencephalography (EEG), and MEG studies, using a range of experimental paradigms, and a range of typologically distinct languages to support the existence of an early, form-based stage of morpho-orthographic segmentation. Investigation of the post-decomposition stages is considerably sparser and less comprehensive. We review previous studies addressing each of these processing stages below.

### Stage 1: Early Form-Based Morphological Decomposition

It is now well established that the earliest stage of processing any potentially morphologically complex written word involves parsing a string into its constituent morphemes on the basis of relatively low-level form characteristics. There is very robust evidence for this early, automatic, semantics-blind, form-based, morphological parsing in the visual domain, for affixal, derivational morphology, in a substantial literature using the visual masked priming paradigm, either on its own [see Rastle and Davis (2008) for a review of 19 studies, and subsequent work such as Diependaele et al. (2009) and Dominguez et al. (2010)], or in concert with EEG and MEG recordings (Lavric et al., 2007; Morris et al., 2007, 2008; Marslen-Wilson et al., 2008; Lehtonen et al., 2011; Royle et al., 2012). In these masked priming studies, transparently related prime-target pairs like *teacher-teach* and pseudo-related pairs such as *brother-broth* (where a single-morpheme prime can be segmented into constituents which are formally identical with an existing stem and affix) are compared with form–overlap pairs such as *brothel-broth* (where the degree and position of orthographic overlap are identical to the other pairs, but the prime ends with a string that is not an affix in the language). Primes are presented for very short durations (typically between 30 and 50 ms), and related prime–target pairs are contrasted with unrelated pairs such as *warfare-broth*. Behaviorally, both the transparently related, and the pseudo-related priming conditions are associated with significantly faster lexical decision response times than the unrelated condition, and in the EEG studies, the two related priming conditions are associated with modulations in early evoked responses [see Morris and Stockall (2012) for a review].

Evidence for early, automatic, form-based decomposition of all potentially morphologically complex strings from the behavioral and ERP masked priming studies is complemented by a convergent line of evidence using single word reading studies and MEG to investigate both the temporal and spatial characteristics of this decomposition process. Zweig and Pylkkänen (2009), in a landmark study, demonstrated that the M170 response component, a bilateral evoked response peaking approximately 170 ms after the onset of visually presented stimuli, and originating in fusiform gyrus (the “Type II Activity” in Tarkiainen et al., 1999) is differentially sensitive to whether a wordform can be exhaustively parsed into a stem and affix. Subsequent investigation of this evoked response has used correlational analysis techniques to show that the M170 reliably tracks a range of form-based cues to segmentation, including affix frequency and stem:whole word transition probability [i.e., the ratio between stem frequency and whole word frequency, a variable argued by Hay (2001) to index decomposability] (Solomyak and Marantz, 2010; Lewis et al., 2011; Gwilliams and Marantz, 2018) as well as stem frequency (Gwilliams et al., 2016) but not whole word lexical properties.

Across these studies, stem:whole word transition probability in particular was found to be a reliable index of whether the visual system considers a word to be morphologically complex. Gwilliams et al. (2016) developed and tested a functional localizer for the M170 response and found that more activity is elicited when a suffixed word has high transition probability. This response was found between 150 and 200 ms in left fusiform gyrus, thus corroborating previous studies.

### Stage 2: Post-decomposition Processes

A variety of studies using fMRI, EEG, and MEG have provided evidence for the existence of the post-decomposition stage, in which the decomposed parts are recombined, mostly focusing on *lexeme lookup* and on the evaluation of semantic well-formedness (*composition*) and less so on subcategorization-based *licensing*.

#### Lexeme Lookup

Activation of stored lexical items has been robustly associated with the evoked N400/M350 response component. This response originates in relatively anterior portions of the temporal lobe, starting at about 200 ms post-stimulus onset (PSO) and peaking between 300 and 400 ms. Previous MEG studies focusing on morphologically complex stimuli have found that morphological family size and family frequency both modulate this response (Pylkkänen and Marantz, 2003; Solomyak and Marantz, 2010), and that this response is associated with a lemma (stem) frequency effect, distinct from later effects of whole word frequency (Solomyak and Marantz, 2010; Fruchter and Marantz, 2015) [see Lau et al. (2008) for a review of further findings associating the N400 response with lexical access].

#### Licensing Based on Grammatical Category

A number of studies have investigated the temporal and spatial dynamics of early access to grammatical category. Friederici (2002)’s serial model of language processing, for instance, argues that the syntactic category of a word is identified 100–300 ms after stimulus presentation. This claim is supported by ERP/EEG studies (Hahne and Friederici, 1999; Hahne and Jescheniak, 2001) using a violation paradigm, in which the response to either a free-standing function word or bound inflectional affix which is inconsistent with the syntactic context is compared to the response to a grammatical, consistent word in the same context. The ERP studies identify an early left-anterior negative ERP response (ELAN) in the inferior portion of the superior temporal gyrus (STG) peaking at about 250 ms in response to these grammatical category mismatches^1^. MEG studies using a similar paradigm (Dikker et al., 2009) identify an even earlier response peaking around 100 ms PSO, originating bilaterally in occipital cortex.

Linzen et al. (2013) used a single word reading paradigm, with no predictive syntactic context, and investigated the effect of subcategorization frame entropy using MEG. This variable quantifies the uncertainty over the possible syntactic phrases a verb can take [a combined measure of the number of possible frames a verb can be followed by, and the extent to which their distribution is balanced, reflecting the degree of uncertainty about the syntactic category of the verb’s complement (del Prado Martín et al., 2004)] as its complement and was found to significantly correlate with activity in the anterior temporal lobe (ATL), from 200 to 300 ms. This result was later replicated (Sharpe et al., 2019). Building on the findings of these studies, King et al. (2016) used noun/verb (N/V) entropy as a variable to investigate whether the syntactic category features of the word itself also modulate the activity in the ATL in this early time window. High N/V entropy occurs when the probability of using a word like *scoop* as a verb is similar to the probability of using it as a noun, while low N/V entropy occurs when the probabilities of the two options are very different. King et al. (2016) report a significant correlation between N/V entropy and activity centered at about 220 ms in the ATL and argue that it indexes a point in lexical access at which the syntactic properties of the stem are assessed.

#### Composition and Well-Formedness

Fruchter and Marantz (2015) succeed in establishing spatially and temporally distinct profiles of at least two stages of recomposition: *lexeme lookup* and *combination*, in a single-word-reading MEG study. The study involved well-formed morphologically complex English words that varied along two dimensions, each of which was argued to be a probe for the two distinct processing stages, and a simple lexical decision task. The variable used to investigate lexeme lookup was derivational family entropy (a statistical measure of the distribution of lexical frequencies within the derivational family of the stem, del Prado Martín et al., 2004) and the variable used to investigate combination was a novel variable they called *derived semantic coherence*, which takes into account the discrepancy between the actual whole word frequency of a complex word, and its predicted frequency given the frequencies of its constituents, modulated by English morpho-phonological restrictions. They found a region in middle temporal gyrus (MTG) that showed earlier sensitivity to derivational family entropy from 241 to 387 ms PSO and later sensitivity to whole word surface frequency from 431 to 500 ms PSO; and a region in orbitofrontal (OF) cortex that showed sensitivity to derived semantic coherence in a more sustained response (354–500 ms PSO).

Whiting et al. (2014) combined a single word, brief presentation design with MEG recordings, and compared genuinely complex words like *teacher* with pseudo-complex words like *corner*. Pseudo-complex words evoked the same processing profile as genuinely complex forms in initial processing stages [morpho-orthographic decomposition in left inferior temporal gyrus (ITG) and left fusiform gyrus between 150 and 230 ms PSO]. Morphological complexity effects plausibly related to lemma access in left MTG emerged between 300 and 360 ms PSO, with pseudo-complex forms evoking greater activation. This dissociation between effects of morphological complexity in the MTG between 300 and 360 ms and effects of whole word lexicality in posterior STG at a later time window is the same pattern observed by Fruchter and Marantz (2015), and consistent with a model in which initial lexical access is morphologically mediated (based on activating the stems and affixes identified in the form-based decomposition analysis), with whole word features only becoming relevant at a later stage.

The difference between the two studies stems from the types of stimuli/variables used which allow us to approach the *recomposition* stage from two different angles. Fruchter and Marantz’s (2015) stimuli (existing derived words) evoked OF activity showing sensitivity to derived semantic coherence while Whiting et al.’s (2014) use of pseudo-complex forms evoked left ITG/fusiform gyrus. Pseudo-complex forms which are parsed into potential morphological constituents involve a mismatch between the meaning and grammatical features that are generated for them by combining these morphological constituents and the familiar, non-compositional meaning actually associated with the whole word. A morphological parse of *corner* would yield the derived agentive noun “someone who corns” or the comparative adjective “more cornlike,” while a whole word lookup yields the noun meaning “a place or angle where two sides or edges meet” (or various related meanings) or the verb meaning “force (a person or animal) into a place or situation from which it is hard to escape.” Crucially, the problem with a morphological analysis of “corner” is not that it is in and of itself ungrammatical or semantically illformed, but rather that it conflicts with the stored whole word representation activated by the exact same string. This result is therefore not in direct conflict with the association of semantic illformedness with OF activation. Whiting et al. (2014) attribute the effects they observe to “top–down feedback processes” (p. 259) analogous to garden-path effects observed in sentence processing, and not directly to morphological composition.

Neophytou et al. (2018) presents the first attempt to systematically investigate all the processing stages proposed in Schreuder and Baayen’s (1995) meta model. The study reports MEG recordings from a single word reading experiment in which well-formed, grammatical morphologically complex words are contrasted with two types of deverbal pseudowords in Greek which violate either the subcategorization frame of the stem + suffix (*licensing*) or the argument structure properties of the two (*composition*). That is, brain activation was recorded for categorical violations of the type ^∗^*potam-imos* “^∗^river-able,” a violation of the syntactic category requirements of the affix, and for argument structure violations of the type ^∗^*gela-simos* “laugh-able,” a violation of argument structure requirements. Neophytou et al. (2018) found:

(A) For the grammatical words: (i) an early effect of *decomposition* within the first 200 ms PSO in the left fusiform gyrus, and (ii) a dissociation between early (200 and 400 ms) stem frequency effects (*lexeme lookup*) and later (>400 ms) surface frequency effects (*whole word processing*) in left temporal lobe.
(B) For the pseudowords: (i) larger amplitudes for category violation items than argument structure violation items in the temporal lobe from 200 to 300 ms (*licensing*), and (ii) larger amplitudes for argument structure violation items than for category violation items in OF cortex between 300 and 500 ms (*composition*).

Taken together, these recent papers provide a consistent “map” of the complete process of processing a morphologically complex word, from initial form-based segmentation through lexical activation, syntactic licensing, and semantic interpretation. The Neophytou et al. (2018) results from Greek further suggest that the spatial and temporal dynamics of this process are very similar across different languages. However, these results, and most of the earlier MEG, EEG, and fMRI results they build upon, all involve suffixed words.

### But What About Prefixes

Taft and Forster’s (1975) experiments, and the model they developed as a result, were focused on testing the strongest version of their affix-stripping hypothesis, namely that a word like *rejuvenation* is recognized by stripping off the affixes *re-*, *-ate*, and *-ion*, and accessing the stored lemma “juv,” which never occurs as a free-standing word of English. Nonwords like “juvenate,” which contain such a bound stem lemma should be more difficult for speakers to correctly reject as nonwords than nonwords like “luvenate” which do not contain any bound stem lemma. As they explain, for cases like “luvenate,” the item can be identified as a nonword after a search in the lexicon is unsuccessful, but for cases like “juvenate” a lexical representation of the stem would be found. Correctly determining that despite this stem representation, the string “juvenate” is not a word, would thus require additional processing.

**FIGURE 1:**
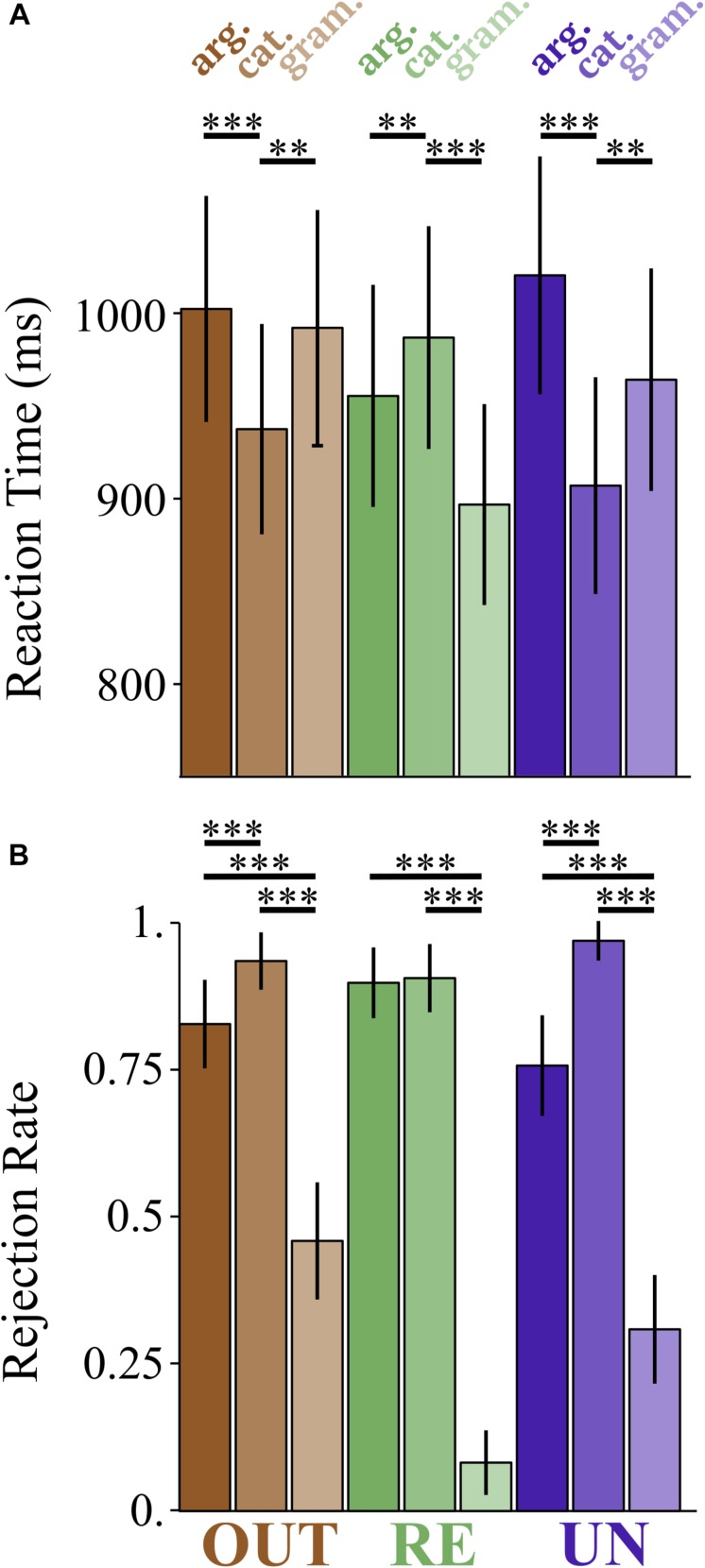
Effects of grammaticality, prefix, and violation type on lexical decision responses. **(A)** Reaction time (ms). **(B)** Rejection rate. Response is coded as 0 for accepted trials, 1 for rejected trials. ^∗∗^*p* < 0.01 and ^∗∗∗^*p* < 0.001.

Taft and Forster (1975) make an important distinction between words like *rejuvenate*, in which the affix *re-* contributes a restitutive meaning to the whole word, just as it does in cases where it attaches to a free standing verb like *refill*, and words like *repertoire*, where the string “re” makes no contribution to the whole word meaning or its grammatical properties (*repertoire* is a noun, whereas r*e-* prefixation results in verbs). Although initial, form-based affix stripping would still apply to words like *repertoire* in their model (see Taft and Forster, 1975, p. 644; Figure 1), the stem “pertoire” is not found in the lexicon, so the morphological analysis is abandoned and a whole word search (step 4 in Figure 1) is initiated.

Taft and Forster’s (1975) Experiment 1 contrasts the processing of strings like “juvenate” and “pertoire,” and finds, consistent with predictions, that strings containing real bound stems like “juventate” are slower to be rejected than strings containing pseudo-stems like “pertoire.” Real stem items were also less likely to be rejected as nonwords than pseudo-stem items, again consistent with a model in which bound stems like “juv” are listed in the lexicon. In their Experiment 3, they compare the same two categories of non-words, this time with inappropriate prefixes added, as in “dejuvenate” or “depertoire.” In the real stem condition, *de-* is stripped (1), the stem “juv” is identified (2), the prefix+stem combination is assessed and rejected (3), whole word search is initiated (4), and eventual failure leads to a NO decision. In the pseudo stem condition, processing proceeds straight from steps 2–4, with no step 3 required. The additional processing step for real word stems is predicted to lead to a processing slow down, and indeed, the dejuvenate type stimuli are processed more slowly than the depertoire type items.

Pseudostem prefixed items are not directly compared with genuinely prefixed items in the 1975 studies, but in follow up work, Taft (1981) found that pseudo-prefixed words, which begin with an orthographic string that is identical to a real prefix, but cannot plausibly be parsed as morphologically complex (e.g., re-cipe) are recognized as real words more slowly than bound-stem prefixed words, where the prefix and stem are plausibly analyzed as units attested elsewhere in the language [e.g., re-sume (compare re-mit, con-sume)]. In a similar study in Dutch, Bergman et al. (1988) compare prefixed, pseudo-prefixed, and unsegmentable words. Pseudo-prefixed words were consistently accepted significantly more slowly than either genuinely prefixed words or unsegmentable words in a series of lexical decision experiments, which they argue suggests that “the recognition of pseudoprefixed items is substantially impaired by a failed attempt at decomposition.” (p. 56).

The most serious detractor from the prefix stripping view is Schreuder and Baayen’s (1994) “Prefix Stripping Re-revisited,” which, by comparing lexical statistics on prefixation and pseudo-prefixation in English and Dutch, shows that the addition of a prefix stripping module to a serial search model (as in Taft and Forster, 1975) leads to a substantial decrease in its processing efficiency in English and Dutch, because both languages have a large number of words like “repertoire” involving pseudo-prefixation (e.g., they identify 98% of all words beginning with “de” as pseudo-prefixed according to their criteria), and pseudo-prefixation triggers costly backtracking in the serial Prefix Stripping model. Given that the initial articulation of this model was motivated by the proposal that a morphologically accessed lexicon would be substantially more computationally efficient than a whole word listing lexicon, Schreuder and Baayen’s (1994) calculations pose a serious challenge. The article concludes that it is highly improbable that prefix-stripping is an automatic early stage in lexical processing as envisioned by Taft and Forster (1975), stating “prefix stripping cannot be motivated on the basis of efficiency arguments” (p. 370).

Much subsequent work investigating the predictions of the Affix Stripping model relies on priming paradigms rather than single word processing studies. Perhaps the most influential such study is Marslen-Wilson et al. (1994), who directly compares prefixation and suffixation in a series of cross-modal priming experiments and find robust priming from both prefixed and suffixed primes to their stems, even where the relationship is partially obscured by phonological opacity (e.g., serenity∼serene). These results are consistent with a version of the model in Taft and Forster (1975) in which both prefixes and suffixes are stripped at the earliest stage of processing, and only stems are stored in the mental lexicon. Other studies find equivalent priming effects for prefixed and suffixed words (e.g., Giraudo and Voga, 2013; Beyersmann et al., 2016), or robust priming for prefixed words in masked priming paradigms (Forster and Azuma, 2000; Chateau et al., 2002; Nikolova and Jarema, 2002; Diependaele et al., 2009; Kazanina, 2011).

However, differences between the processing of prefixed and suffixed words have also been found. Meunier and Segui (2002), replicating the design of Marslen-Wilson et al. (1994) in French, find the same results for prefixes and suffixes in all cases, except that while prefixed words prime their stem correlates regardless of phonological opacity, suffixed words only prime their stems if they are transparently phonologically related. In a similar vein, Giraudo and Grainger (2003) find that prefixed primes facilitate target recognition latencies in French (e.g., prénom–préface), but that suffixed primes do not. Indeed several studies find this pattern of a more robust priming effect for prefixed than suffixed words (see also Grainger et al., 1991; Feldman and Larabee, 2001). Diependaele et al. (2009) suggest that these differences may be attributed to a “beginning-to-end” sequential bias, in which prefixes are more salient, and more rapidly detected than suffixes (see also Bergman et al., 1988; Libben, 1994; Giraudo and Grainger, 2003).

Interestingly, a range of other studies find differences between prefixed and suffixed words that are not so easily reconciled with the Affix Stripping Model claim that all affixes are processed via the same mechanisms. Colé et al. (1989) find that cumulative root frequency determines the latencies to suffixed words but not to prefixed words, suggesting an asymmetry in the storage or retrieval of prefixed vs. suffixed stems. Beauvillain (1996) investigating the processing of affixed words in sentential context using eye-movement measures finds that cumulative root frequency affects first-fixation duration on suffixed words and second-fixation duration on prefixed words, but that surface frequency has no effect on these early measures for either affix type. These results suggest that both prefixed and suffixed words are parsed into their constituents early in visual word recognition, but that suffix stripping may be faster than prefix stripping. Kim et al. (2015) investigated the processing of prefixed and suffixed real and novel words in Korean in a masked priming experiment, and found that while suffixed primes facilitated responses to their stem targets regardless of the lexicality or interpretability of the primes, prefixed primes significantly affects target responses only when they were real words, and not when they were either interpretable or noninterpretable prefixed pseudowords. They conclude that these results are consistent with a model in which suffixes are stripped prelexically, but prefixed words are only decomposed after lexical access has occurred. Beyersmann et al. (2015) also find an asymmetry between prefixes and suffixes in a letter detection task in French: target letter detection was slower in suffixes than in non-suffix endings, but no such effect was found for prefixes, which the authors interpret as evidence that only suffixes are rapidly stripped from stems and made unavailable to fine-grained orthographic processing.

Overall, the psycholinguistic research on prefix and suffix stripping presents a mixed picture: many studies reporting results consistent with both prefixes and suffixes being subject to the same early, form-based, affix stripping parsing mechanism, but many other studies calling these results into question. The challenges include both computational modeling work arguing that purely form-based prefix stripping would lead to mis-parsing far more often than it would lead to successfully detecting a real stem, and processing studies failing to find affix-stripping effects for prefixes.

Curiously, despite the theory-critical status of pseudo-affixation of the “repertoire” type, it has received remarkably little attention in the literature, with far more research focusing on words like “release” or “department,” which can be exhaustively parsed into real stems and affixes, though not transparently semantically recomposed. However, the one MEG experiment to investigate prefix processing, Zweig and Pylkkänen (2009), did include such items. They conducted one experiment with suffixed forms (genuine complex form: *teacher*, unsegmentable monomorph: *straight*, pseudo-affixed: *winter*), and one experiment with prefixed forms (genuine complex form: *refill*, unsegmentable monomorph: *straight*, pseudo-affixed: *resume*). A significant increase in M170 peak amplitude for the genuinely complex forms compared to both the unsegmentable and pseudo-affixed forms was found bilaterally for the prefix experiment and only in the right hemisphere for the suffix experiment, with no differences between the unsegmentable and pseudo-affixed words. Zweig and Pylkkänen (2009) consider various explanations for the differences in lateralization between the two affix types but conclude “every account has to appeal to decomposition in some way, since that is the only stimulus factor that can explain the right-hemisphere effect obtained in both Experiments” (p. 433). Critically, pseudo-stem words like *winter* and *resume* pattern like the unambiguously monomorphemic items, suggesting “affix stripping” does not apply in all cases (contra the assumptions of Schreuder and Baayen, 1995).

Gwilliams and Marantz (2018) followed up on this result, using MEG to compare the processing of pseudo-words of the *winter* type with words like *excursion* (“excurs-” + ion) which contain a stem that is otherwise unattested in the language, but where the affix makes a predictable, transparent contribution to meaning and grammar. *Excursion* denotes a verbal action just as transparently complex *-ion* nominals like *rebellion* do. This is not the case for winter-type words, which do not follow any regular morpho-syntactic rules (*winter* is neither “someone who wints” or “more wint than X”). Gwilliams and Marantz (2018) found evidence that the visual system uses morpho-syntactic rules to avoid incorrectly parsing words into affixes and non-existent stems (e.g., wint-er, re-ad). Perhaps, then, the system uses more than the mere presence of an affix and statistical cues such as transition probability in order to parse morphologically complex forms. This suggests that the system would not fall victim to garden-path-parses as often as Schreuder and Baayen’s (1994) computations suggest, and therefore that grammatically constrained prefix stripping may yet be a viable model.

The inconclusive picture offered by the processing literature, the discovery by Gwilliams and Marantz (2018) that early, form-based affix stripping appears to be grammatically constrained in a way that may obviate the strong computational argument against prefix stripping, and the recent multi-stage mapping research by Neophytou et al. (2018) and Fruchter and Marantz (2015) make the time ripe for re-revisiting prefix processing.

The current experiment takes the design and analysis protocols from Neophytou et al. (2018) but substitutes English prefixed words and pseudowords for the Greek suffixed materials in that study. The experiment was run in tandem with Gwilliams and Marantz (2018), which reports clear left lateralized fusiform gyrus sensitivity to stem:whole word transition probability from 150 to 180 ms for suffixed words. Given the results of Zweig and Pylkkänen (2009), and the results of the many decades of behavioral work reporting similar processing profiles for prefixed and suffixed forms, we would expect to find that prefixed items evoke the same decomposition effects as Gwilliams and Marantz (2018) and Neophytou et al. (2018), and the same pattern of recomposition effects as Neophytou et al. (2018), as summarized above. However, unlike a behavioral processing experiment, or even an ERP study investigating the modulation of a single response component, MEG, and the materials and design employed in this study enable us to investigate a whole set of spatially and temporally distinct evoked responses, allowing the possibility of finding that prefixed words are processed similarly to suffixed words at some stages, but differently at others.

## Materials and Methods

### Materials

The test items consisted of pseudowords with grammatical category (Cat) or argument structure (ArgStr) violations, and grammatical words, with one of the following three prefixes: *out-*, *un-*, and *re-*. Each of these three prefixes has distinct category and argument structure restrictions as summarized in Table 1.

**TABLE 1:** Summary of category and argument structure restrictions on re-, un-, and out-prefixation.

**Prefix**	**Category restriction**	**Argument structure restriction**
Out-	✓ verb: *outrun* × adj: *outblue* × noun: *outcat*	Attaches to intransitive verbs to create a transitive verb (*^∗^Ari dances Zi* →✓ *Ari outdances Zi*), so cannot attach to obligatorily transitive verbs (*^∗^outmurder*) (Irube, 1984; Marantz, 2009; Alexiadou et al., 2014; Tolskaya, unpublished)
Re-	✓ verb: *refill* × adj: *rehard* × noun: *rehat*	Attaches to verbs that can take a direct object and which denote a result state (*rebraid*), so cannot attach to intransitive verbs (*^∗^relaugh*), or ditransitives (*^∗^reput*) (Marantz, 2007; Alexiadou et al., 2014)
Un-	✓ verb: *unroll* ✓ adj: *unkind* × noun: *untea*	Attaches to verbs which denote a reversible change of state (*unbutton*) so cannot attach to psychological verbs or statives (*^∗^unknow*, *^∗^unannoy*) (Sawada, 1995; Horn, 2005; Pylkkänen et al., 2009b)

*^∗^indicates an ungrammatical sentence or word.*

The prefix re- was included both because it has figured so prominently in the previous psycholinguistic and neurolinguistic research on prefixation processing, especially in Schreuder and Baayen’s (1994) argument that prefix stripping would be computationally inefficient, but also because it has been well studied in formal linguistic research (Marantz, 2007; Alexiadou et al., 2014), and thus has a well described grammar. Verbal *un-* has also previously been investigated in neurolinguistic (Pylkkänen et al., 2009b), psycholinguistic (de Almeida and Libben, 2005; Pollatsek et al., 2010), and formal linguistic (Sawada, 1995; Horn, 2005) research, though to a lesser extent than *re-*. *Out-* has received no previous attention in any processing literature we are aware of and it is also under-investigated in the formal linguistic literature: apart from Irube (1984), prefixal *out-* has only been discussed in unpublished conference presentations (e.g., Marantz, 2009; Ahn, 2015), or in passing, in the context of discussions of verbal argument structure (e.g., Keyser and Roeper, 1984; Rappaport Hovav and Levin, 2002; McIntyre, 2003; Beavers and Koontz-Garboden, 2012) but was included as a potentially interesting contrast to *re-* and *un-*. Unlike these prefixes, which require the verbal constituent they attach to to take an internal argument that undergoes a change of state (e.g., repaint the wall, unfold the paper) and therefore attach to unaccusative/accomplishment verbs, *out-* adds an extra argument to a verbal predicate (Kai sang → Kai outsang Zi) and thus attaches to unergative/activity verbs or verbs which are at least optionally intransitive.

For all three prefixes, we compare three critical conditions, all consisting of the prefix attached to a monomorphemic stem:

(a) grammatical, attested, semantically transparent: e.g., *refill*, *unplug*, *outthrow*;
(b) category violating stem: e.g., *reblue, uncat, outsad*;
(c) category licit, but argument structure violating stem: e.g., *relaugh, unthink, outmurder*.

Material selection began with identifying as many possible candidates for each condition, and then applying a series of constraints to select the materials used in the study. These processes, described below, resulted in 31 items per condition for *out-* and *re-* (31 grammatical, 31 category violating, 31 argument structure violating), and 35 items per condition for *un-*. Within each prefix, items were matched for average length and stem frequency across conditions using the English Lexicon Project corpus (Balota et al., 2007). Stimulus characteristics are in Table 2.

**TABLE 2:** Mean length and log stem frequencies for critical stimulus items.

**Prefix**	**Condition**	**Ave.length**	**Ave.log.stem.freq**
Out-	Grammatical	4.74	3.326
	Category violating	4.84	2.890
	Argument structure violating	5.13	2.921
Re-	Grammatical	5.42	3.173
	Category violating	5.42	2.564
	Argument structure violating	5.39	2.153
Un-	Grammatical	4.66	2.917
	Category violating	4.54	2.712
	Argument structure violating	4.97	3.222

### Material Selection

Grammatical candidates were chosen by selecting all the monomorphemic stem *out-*, *un-*, and *re-* prefixed items in the SUBTLEX database (Brysbaert and New, 2009).

Category violating stem candidates were selected by choosing all the monomorphemic, 100% adjectival (*out-*, *re-*), or 100% nominal (*un-*) stems in the SUBTLEX-POS database. Noun rather than adjective stems were chosen for *un-* because the other *un-* can attach to adjective stems (e.g., unkind).

Argument Structure violating candidates were identified using different procedures for each prefix, due to the differences in the nature of the restrictions. For *out-* candidates were found using the Unified Verb Index database at the University of Colorado. The Unified Verb Index is a system which merges links and web pages from four different natural language processing projects: VerbNet, PropBank, FrameNet, and OntoNotesSenseGroupings (Baker and Ruppenhofer, 2002). It includes 8537 verbs, all annotated for syntactic/semantic frame (each project uses slightly different ontologies), and for their Levin Class (Levin, 1993). We used Levin Classes to identify verbs that were obligatorily transitive (i.e., had no intransitive occurrences in any of the UVI sub-databases). For *un-*, items were chosen from Levin (1993) to cover a range of verb types which do not satisfy the requirement of denoting a reversible change of state to an affected argument (e.g., stative verbs, unergatives, psych-verbs, etc.). Only stems which have a >60% verb dominance score in the SUBTLEX-POS database (Brysbaert et al., 2012) were considered. Candidate items were googled, and excluded if they were found to be attested, in a coherent sentence (machine generated word lists, etc. were ignored). For *re-*, the argument structure restriction prohibits verbs that have too many arguments (ditransitives, e.g., ^∗^reput), verbs that have too few (unergatives, e.g., ^∗^relaugh), and verbs that denote states (e.g., ^∗^relove), activities (e.g., ^∗^rewalk), and perceptual experiences (e.g., ^∗^resee, ^∗^refear). We chose to focus on unergative verb stems. All monomorphemic verb stems with NULL as their most frequent verb frame in the VALEX subcategorization database (Korhonen et al., 2006) were considered. Candidate stems were excluded if they occurred with *re-* prefixation in CELEX or the ELP. The remaining candidates were manually assessed for their well-formedness in two frames designed to distinguish unergative and unaccusative verbs:

(a) The frame “a recently VERB-ed X,” where X had to be an attested subject of the verb (COCA was used to identify a subject). If a verb was acceptable in this frame, with an eventive interpretation, it was excluded from further testing. This test should only be good with unaccusative, internal, affected argument having verbs (Alexiadou and Schäfer, 2011).
(b) The frame “VERB-ed a _____ COGNATE-NOUN,” where the blank slot was filled with a degree adjective. If the verb was acceptable in this frame, it survived as a candidate item (Levin and Rappaport Hovav, 1995; Hale and Keyser, 2002).

To ensure an equal number of grammatical and ungrammatical trials, grammatical filler items beginning with the same three prefixes needed to be added. Because the critical, morphologically simple-stem, grammatical *un-* items essentially exhaust the set of such items in English, additional grammatical *un-* prefixed items had to have complex, derived verb stems (*unflatten*, *unthinking*). Since such three morpheme items have not previously been investigated in any MEG experiment focused on morphological decomposition and recomposition, it was decided to make a virtue of necessity and also include three morpheme, derived-verb-stem grammatical fillers for the other prefixes, although these items are not analyzed here. A total of 140 such grammatical three-morpheme items were included in the materials set, along with additional ungrammatical fillers (66), and grammatical monomorphemic fillers (23). In total, the experiment included 260 grammatical targets + 260 ungrammatical targets for a total of 520 target items (202 or 39% r*e-*; 93 or 18% *out-*; and 225 or 43% *un-*). A complete set of critical and filler items is available as the Supplementary Material.

### Participants

Twenty-five participants (10 male, mean age 21.8 years, *SD* = 6.52) from among the NYU Abu Dhabi community participated in the experiment. All participants were right-handed, with normal or corrected-to-normal vision, and spoke English as a first language.

### Procedure

Presentation^®^ software (Version 18.0, Neurobehavioral Systems, Inc., Berkeley, CA, United States^2^) was used as the presentation platform. The stimuli were projected onto a screen that was located approximately 85 cm away from the participant.

Prior to the main experiment, the abridged functional localizer reported in Gwilliams et al. (2016, Experiment 3) was run on all subjects. This localizer consists of a 6-min long sequence of symbols, letters, and words presented in four levels of Gaussian noise. Participants were instructed to focus on the pictures and avoid blinking to minimize MEG data artifacts. No active response to the stimulus was required. See Gwilliams et al. (2016) for a more detailed discussion of the features of this localizer.

The main task of the experiment was a visual lexical decision task, with simultaneous MEG data recording. Each trial began with a fixation cross (“+”) for 400 ms, followed by a single item that stayed on the screen until the participants gave a response, or for a maximum of 2 s. Participants were instructed to indicate whether the item was a real word in English by pressing one of two buttons with their left hand. In order to familiarize the participants with the task, we included a short practice with eight items at the beginning of the session, none of which was included among the test items. For the practice, if the participant gave a wrong answer a red cross appeared. During the actual test session, no feedback was provided. The items were fully randomized and each participant received a unique randomization. The experiment consisted of four blocks and lasted around 20 min.

A 208-channel axial gradiometer whole-head MEG system (Kanazawa Institute of Technology, Kanazawa, Japan) was used to record the data continuously, at a sampling frequency of 1000 Hz. The data were filtered during acquisition between 0.1 and 200 Hz. The head of every subject was digitized prior to entering the magnetically shielded room using a hand-held FastSCAN laser scanner (Polhemus, VT, United States). The head position during the experiment was determined using coils attached to predefined anatomical regions. The head scan and the coil measurements were then used for the co-registration process.

Each recording session also included a second MEG experiment, investigating the processing of suffixed words. These results are reported in Gwilliams and Marantz (2018). The order of the two experiments was counterbalanced across participants. Note that only 24 of the 25 participants were analyzed in Gwilliams and Marantz (2018), due to noise issues with one participant.

### Data Analysis

#### Behavioral Data

Reaction times (RTs) and yes/no responses were recorded for every trial. Coefficients were estimated with linear mixed-effects models (Baayen et al., 2008), using the lmer function of the lme4 package in R (Bates and Maechler, 2009) for the RT data, and the glmer function of the lme4 package in R to fit logistic regression on the acceptance rate data. The fixed effects in the maximal model were Condition Type (i.e., Cat, ArgStr, or grammatical) and Prefix Type (i.e., *out-*, *re-*, or *un-*). By-subject slopes for all model terms, as well as by-subject and by-item intercepts were also fitted as random effects. Model comparisons were then performed by extracting one fixed term at a time, beginning with the random effects structure (Barr et al., 2013). Condition was coded into two orthogonal contrasts: Grammaticality, which compared grammatical items to the average of all the ungrammatical items, and Violation Type, which compared Argument Structure violations to Category violations.

Responses deviating more than 2.5 standard deviations from the mean response for each participant × condition were excluded from analysis, resulting in the rejection of 5% of all trials. Response times were log transformed to minimize skew. For the response time analysis, only trials that were correctly accepted (grammatical items) or correctly rejected (category and argument structure violation items) were analyzed.

#### MEG Data

MEG raw data were first noise reduced using three gradiometer reference channels located away from the participant’s head, utilizing the Continuously Adjusted Least Squares Method (CALM; Adachi et al., 2001) in the MEG160 software (Yokogawa Electric Corporation and Eagle Technology Corporation, Tokyo, Japan). The noise-reduced data were further preprocessed and analyzed with MNE-Python (Gramfort et al., 2013, 2014) and Eeelbrain^3^. Independent component analysis (ICA) was conducted to remove components related to specific noise patterns, while additional artifact rejection was performed through manual inspection of the data. The data were low-pass filtered at 40 Hz and epoched from −200 to 600 ms, relative to the beginning of the stimulus. Structural MRIs were reconstructed by scaling and orienting the Freesurfer average brain (CorTech Labs, La Jolla, CA, United States and MGH/HMS/MIT Athinoula A. Martinos Center for Biomedical Imaging, Charleston, MA, United States) to each participant’s head shape. A source space consisting of 5124 vertices, equally split into the two hemispheres, was generated on each reconstructed surface. Once we baseline corrected the data using a −200–0 ms pre-stimulus interval, the boundary-element model (BEM) method was utilized on the activity at each of the vertices to compute the forward solution. Using the forward solution and the grand average of the data for all trials within each subject, the inverse solution was calculated.

Following Gwilliams et al. (2016), Gwilliams and Marantz (2018), and Neophytou et al. (2018), the inverse solution was calculated using signed fixed orientation for the source estimates. This means that the direction of the current normal to the cortex was defined, and the dipoles were projected perpendicular to the cortical surface, estimating activity from the magnitude of the current dipole normal to the cortex. The signed normed estimates were transformed into noise-normalized dynamic statistical parameter maps (dSPMs; Dale et al., 2000), using an SNR value of 3 for ANOVA analyses and a value of 2 for the regression analyses. Although polarity in MEG data is still far from being fully understood, opposite polarity activities may reflect discrete response components (for further discussion, see Gwilliams et al., 2016).

##### The Tarkiainen localizer

The functional localizer was used in order to identify the occipito-temporal response that corresponds to the M170 component – the locus of expected decomposition processes. See Gwilliams et al. (2016) for analysis details.

##### The morphological processing experiment

The statistical analysis of the data varied depending on the question each analysis was aiming to answer, as well as on the region of interest (ROI). Spatio-temporal tests were run on anatomical ROIs, while mixed effects models were run on functional ROIs (fROIs).

The fROIs were validated using an orthogonal dataset (Gwilliams and Marantz, 2018), and provide a specific hypothesis about which locations in the brain will show experiment-relevant modulations. In this case, we averaged activity within the spatial extent of the fROI and submitted this average activity to statistical analyses. Linear mixed-effects model analyses utilized the lmer function of the lme4 package in R. The linear mixed-effects models included the neural activity averaged across both time and space as the dependent variable. Coefficients were estimated with fixed effects of length of the item, number of syllables, log stem frequency, log surface frequency, average bigram frequency, prefix orthographic *n*-gram frequency, prefix morphological *n*-gram frequency, and stem:whole word TP. By-subject random slopes were fitted for all the lexical variables, as well as by-subject and by-item random intercepts. Model comparisons were then performed by extracting one fixed term at a time, beginning with the random effects structure (Barr et al., 2013). Only terms that significantly affected model fit were retained.

By contrast, anatomical ROIs were chosen for effects for which we did not have a functional localizer. Because our hypothesis about the location of the effect is less specific in these cases, we made the anatomical ROIs to be purposefully larger than we expected the response of interest to be, such that we could be confident that the region encompassed the effect. Within this larger ROI, we tested more specifically where in the brain was sensitive to the manipulation of interest using spatio-temporal cluster tests.

Spatio-temporal analyses combined a regression test with permutation cluster tests. In order to test for an effect of Violation Type (category violation or argument structure violation) or Prefix Type (*re-*, *un-*, or *out-*), we ran 2 (Violation Type) × 3 (Prefix Type) ANOVAs, which were combined with permutation cluster tests. For all tests utilizing permutation cluster tests, the *p*-values were corrected for multiple comparisons (*p*_mcc_) over time and space as described by Maris and Oostenveld (2007). The equalize_evoked_count function in eelbrain was used to randomly select equal numbers of trials for each condition in each analysis.

Only trials which were correctly accepted (grammatical items) or correctly rejected (category and argument structure violation items) were analyzed.

## Results

### Behavioral

#### Rejection Rate

Table 3 reports the mean rejection rates for the critical items in this experiment. The pattern we observe in the averages across prefix is exactly as predicted given the results of Manouilidou and Stockall (2014): the grammatical items have the lowest rejection rates, and the argument structure violation items are rejected less often than the category violation items. However, individual conditions were associated with surprising rejection rates: the *out-* prefixed grammatical items are rejected 46% of the time, the *un-* prefixed grammatical items were rejected 31% of the time, and the *re-* prefixed argument structure violation items are rejected at the same rate as the *re-* prefixed category violation items (90%). Statistical analysis using generalized linear mixed modeling, with family = binomial confirms the reliability both of the general pattern and the anomalous individual cells.

**TABLE 3:** Mean rejection rates *and standard deviations* by condition and prefix.

	**Grammatical**		**Category viol**		**Argument structure viol**	
			
**Prefix**	**Mean**	***SD***	**Mean**	***SD***	**Mean**	***SD***
Out-	0.457	0.498	0.941	0.236	0.832	0.374
Re-	0.081	0.272	0.907	0.291	0.901	0.299
Un-	0.310	0.463	0.974	0.161	0.759	0.428
Average	0.282	0.411	0.940	0.229	0.830	0.367

The fixed effects of the initial mixed effect model were Condition, Prefix, and the interaction of Prefix with Condition. Condition was coded into two orthogonal contrasts: Grammaticality, which compared grammatical items to the average of all the ungrammatical items, and Violation Type, which compared Argument Structure violations to Category violations. The variable Prefix was coded with the default treatment contrasts, with *un-* as the baseline, comparing *re-* to *un-*, and *out-* to *un-*. The choice of *un-* as the baseline was arbitrary, as we had no hypotheses leading us to expect differences between prefixes. The dependent variable, response, was coded as 0 for accepted trials, and 1 for rejected trials.

Following Barr et al. (2013), we initially constructed a model with the maximum random effects structure possible, which included a random intercept for item, and a random intercept and slope corresponding to the fixed effects structure for subject (1+(violation.type + grammaticality) ^∗^ prefix| subject), including all correlations. However, the model failed to converge with this effects structure, so the random effects structure was trimmed until the model converged, with the resulting model having only random intercepts by subject and item.

To assess the contribution of each fixed effect, model terms were removed one at a time. Model fit was assessed using chi-square tests on the log-likelihood values to compare different models. The best fitting model (modA), which included interactions between Prefix and Violation Type, and Prefix and Grammaticality, was compared to models in which one or the other of these interaction terms was removed (modB, modC). Removing either interaction term resulted in a reduction of goodness of model fit (Pr(>Chisq) = <2.2*e*−16).

modA = response ∼ (violation.type + grammaticality) ^∗^ prefix.

modB = response ∼ violation.type + (grammaticality ^∗^ prefix).

modC = response ∼ grammaticality + (violation.type ^∗^ prefix).

The summary of the best fitting model is in Table 4.

**TABLE 4:** Summary of best fitting glm for rejection rates.

	**Estimate**	**Standard error**	***z*-value**	**Pr(>|*z*|)**	**MCC**
(Intercept)	2.4972	0.191	13.075	<2*e*−16	^∗^
Violation.type (arg.struc)	1.2846	0.3058	4.201	<2*e*−05	^∗^
Grammaticality (bad)	–2.7049	0.2412	–11.216	<2*e*−16	^∗^
Prefix (re-)	0.1564	0.2163	0.723	0.4695	
Prefix (out-)	0.2602	0.2231	1.166	0.24351	
Violation.type (arg.struc):prefix (re-)	–1.2296	0.4328	–2.841	0.0045	^∗^
Violation.type (arg.struc):prefix (out-)	1.2944	0.4457	2.904	0.00368	^∗^
Grammaticality (bad):prefix (re-)	–2.7791	0.3618	–7.681	1.57*e*−14	^∗^
Grammaticality (bad):prefix (out-)	–1.0425	0.3388	–3.077	0.00209	^∗^

*Effects which survive correction for multiple comparisons are indicated with ^∗^.*

Effects that survive correction for multiple comparisons are indicated with a ^∗^, and are as follows: (a) an effect of violation type (β = −1.2846, *SE* = 0.3058, *z* = −4.201), (b) an effect of grammaticality (β = −2.7049, *SE* = 0.2412, *z* = −11.216), (c) an interaction between Violation Type and Prefix for the contrast *re-* vs. *un-* (*z* = −2.841), such that *re-* prefixed argument structure violation items are rejected more often than *un-* prefixed argument structure violation items, (d) an interaction between Violation Type and Prefix for the contrast *out-* vs. *un-* (*z* = −2.904), such that *out-* prefixed argument structure violation items are rejected less often than *un-* prefixed argument structure violation items, (e) an interaction between grammaticality and prefix for the contrast *re-* vs. *un-* (*z* = −7.681), such that *re-* ungrammatical items are rejected less often than *un-* ungrammatical items, and (f) an interaction between grammaticality and prefix for the contrast *out-* vs. *un-* (*z* = −3.077), such that *out-* ungrammatical items are rejected less often than *un-* ungrammatical items. Figure 1B summarizes this pattern of results.

#### Response Time

Table 5 reports the mean time to correctly accepted (grammatical) or rejected (category and argument structure violation) items in this experiment.

**TABLE 5:** Mean times and *standard deviations* for correct responses to the stimulus items in ms.

	**Grammatical**		**Category viol**		**Argument structure viol**	
			
**Prefix**	**Mean**	***SD***	**Mean**	***SD***	**Mean**	***SD***
Out-	967.14	287.91	902.97	245.94	962.52	274.50
Re-	874.09	239.00	959.46	281.27	916.13	271.60
Un-	949.02	269.30	883.41	263.77	1008.22	312.49
Average	930.08	265.40	915.28	263.66	962.29	286.20

While *out-* and *un-* prefixed items exhibited the expected pattern of a slower response to the argument structure violating items than the category violating items (59 ms slower for *out-*, 125 ms slower for *un-*), the *re-* prefixed items triggered the opposite pattern: the argument structure violating items were responded to 43 ms faster than the category violating items. By contrast, while we expected both ungrammatical conditions to be more slowly responded to than the grammatical items (based on Manouilidou and Stockall, 2014; Neophytou et al., 2018), here this is only true for the *re-* items. *Re-* grammatical items were responded to 85 ms faster than *re-* category violating items, but for *un-* and *out-*, the grammatical items were responded to 66 and 65 ms slower than the category violating items. To test the significance of these numeric differences, we used linear mixed effects modeling.

As for the Response analysis, the fixed effects of the initial mixed effect model were Condition, Prefix, and the interaction of Prefix with Condition. Condition was coded into two orthogonal contrasts: Grammaticality, which compared grammatical items to the average of all the ungrammatical items, and Violation Type, which compared Argument Structure violations to Category violations. The variable Prefix was coded with the default treatment contrasts, with *un-* as the baseline, thus Prefix.1 compares *re-* to *un-*, and Prefix.2 compares *out-* to *un-*.

Following Barr et al. (2013), we initially constructed a model with the maximum random effects structure possible, which in this case included a random intercept for item, and a random intercept and slope corresponding to the fixed effects structure for subject (1 + (violation.type + grammaticality) ^∗^ prefix|subject). Due to convergence issues, this maximal effects structure had to be simplified by removing the violation.type × prefix interaction from the subject slope. Further simplifications were compared to this maximal convergent model using chi-square tests on the log-likelihood values to compare the fit of different models. All additional simplifications of the random effects structure resulted in significant reductions in goodness of fit (GoF), thus all terms were kept in the model.

To investigate the fixed effects, the full structure (mod1) was compared to a model in which the violation.type ^∗^ prefix interaction was removed (mod2). This resulted in a reduction of GoF [χ^2^ = 17.358, *p*(χ^2^) = 0.0001701], and so the full model was kept. A second simpler model, in which the grammaticality ^∗^ prefix interaction was removed (mod3) also had a reduced GoF compared to the full model (mod1) [χ^2^ = 45.195, *p*(χ^2^) = 1.535*e*−10].

mod1 = logrt ∼ (violation.type + grammaticality) ^∗^ prefix.

mod2 = logrt ∼ violation.type + (grammaticality ^∗^ prefix).

mod3 = logrt ∼ grammaticality + (violation.type ^∗^ prefix).

The summary of the best fitting model is in Table 6. *P*-values were estimated with the package LmerTest (Kuznetsova et al., 2017). Effects that survive correction for multiple comparisons are indicated with a ^∗^, and are as follows: (a) an effect of violation type (β = 0.058, *SE* = 0.022, *t* = 2.673), such that the argument structure violation items evoked longer RTs than the category violation items, (b) an interaction between grammaticality and prefix for the contrast *re-* vs. *un-* (*t* = −4.410), such that the *re-* ungrammatical items were responded to more slowly than the *re-* grammatical items, but there was no such difference for *un-*, (c) an interaction between violation.type and prefix for the contrast *re-* vs. *un-* (*t* = −3.928), such that for *re-*, argument structure violation items were responded to more quickly than category violation items, but the opposite was true for *un-*, and (d) an interaction between violation.type and prefix for the contrast *out-* vs. *un-* (*t* = 2.871), such that the magnitude of the difference between the two violation types was larger for *un-* than for *out-*.

**TABLE 6:** Summary of best fitting glm for response times.

	**Estimate**	**Standard error**	***df***	***t*-value**	**Pr(>|*t*|)**	**MCC**
(Intercept)	6.809508	0.02764	27.95	246.363	<2*e*−16	
Violation.type (arg.struc)	0.058453	0.021865	134.44	2.673	0.008441	^∗^
Grammaticality	0.048867	0.025247	40.55	1.936	0.059913	
Prefix (re-)	0.008565	0.015484	69.32	0.553	0.58195	
Prefix (out-)	0.008116	0.015351	64.19	0.529	0.598837	
Viol.type (a.s): prefix (re-)	–0.105134	0.026767	247.66	–3.928	0.000111	^∗^
Viol.type (a.s): prefix (out-)	0.075177	0.026187	252.53	2.871	0.004442	^∗^
Grammaticality (bad) prefix (re-)	–0.125934	0.028556	70.62	–4.41	3.62*E*−05	^∗^
Grammaticality (bad) prefix (out-)	–0.031659	0.028632	57.51	–1.106	0.273455	

*Effects which survive correction for multiple comparisons are indicated with ^∗^.*

Figure 1 plots the results for RT and rejection rate. These plots make it clear that the *re-* ungrammatical items are the outliers compared to the other prefixes for both measures, with an opposite direction effect for response time, and a null difference between violation types for rejection rates.

### MEG Data

#### Functional Localizer

The localizer was used to define a ROI that would capture the M170 response. As detailed in Gwilliams et al. (2016), this response can be captured by comparing responses to letter strings and symbol strings. The comparison between these two conditions yielded two significant spatio-temporal clusters, one from 130 to 160 ms (*p*_mcc_ = 0.007, 55 vertices) and one from 130 to 180 ms (*p*_mcc_ = 0.044, 24 vertices) (Gwilliams et al., 2016). The most significant cluster was chosen for the fROI analyses.

#### Decomposition Analysis: >200 ms in Left Fusiform Gyrus

In the early window from 100 to 200 ms, during which the early, obligatory decomposition process is expected to take place, we used linear mixed-effects models to investigate the effect of a number of lexical variables: item length (in letters), log stem frequency, log surface frequency, prefix orthographic *n*-gram frequency, prefix morphological *n*-gram frequency^4^, and stem:whole word TP. All variables were included as fixed effects and random slopes over subjects. Both the left and the right fusiform gyrus were used as the anatomical ROI, and following Neophytou et al. (2018), the most significant of the clusters Gwilliams et al. (2016) identified in the M170 analysis of the functional localizer was used as an fROI to more accurately identify the M170 response. Model comparisons were then performed by extracting one fixed term at a time, beginning with the random effects structure (Barr et al., 2013). Only terms that significantly affected model fit were retained.

The analysis revealed no effects in the left fusiform gyrus and left fROI, and no effects in the right fusiform gyrus between 100 and 200 ms. However, a *post hoc* linear mixed-effects model run over time and then corrected for multiple comparisons revealed significant effects of length (*p*_mcc_ > 0.001) and stem:whole word TP (*p*_mcc_ = 0.019) from 200 to 220 ms. No other variables were significant.

#### Lexeme Lookup Analysis

##### Stem frequency 200–400 ms left temporal lobe

From 200 to 400 ms we used spatio-tempotal regression analyses to look for effects related to Lexeme lookup in the temporal lobe. The effect of stem frequency was investigated in the inferior temporal lobe for all the grammatical items. The analysis revealed a significant cluster in more ventral parts of the inferior temporal lobe (*p*_mcc_ = 0.049), from 225 to 305 ms.

##### Surface frequency 300–500 ms left temporal lobe

To further investigate the effects related to Lexeme lookup we ran another spatio-temporal regression analysis in the temporal lobe investigating the effect of surface frequency, from 300 to 500 ms. No significant cluster correlating with surface frequency was found. No effects of surface frequency were found in earlier time-windows.

#### Licensing Analysis: 200–300 ms Left Temporal Lobe

**FIGURE 2:**
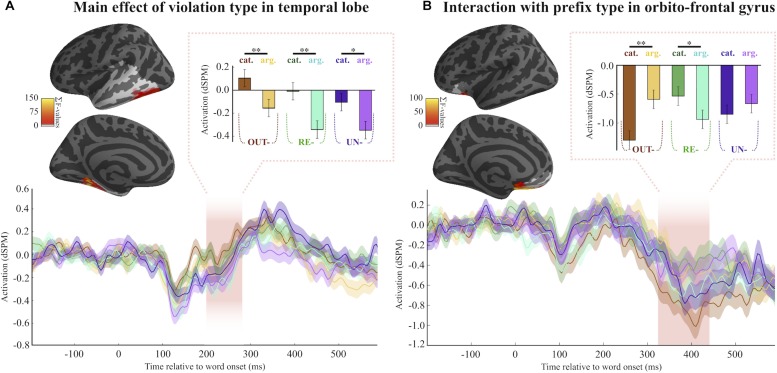
Main MEG results. **(A)** Location and timing of the main effect of violation type. Fsaverage brain shows the location of the spatio-temporal cluster. Every source that was part of the cluster is color-coded with the summed *F*-statistic over time. Pink shaded area refers to the temporal extent of the cluster, which ranged from around 200–300 ms. Barplots correspond to average activity averaged over time and space of the cluster. **(B)** Location and timing of the interaction between prefix type and violation type. Shaded area shows that the effect lasts from around 320–420 ms.

Examination of the 200–300 ms time-window using a spatio-temporal 2 (Cat.Viol vs. ArgStr.Viol) × 3 (*re-*, *un-*, and *out-*) ANOVA in the temporal lobe, where the effects of syntactic licensing are expected to manifest, revealed an effect of Violation Type (Figure 2A). The analysis revealed a significant cluster (*p*_mcc_ = 0.0434) spreading across the ventral and posterior portions of the region we analyzed. Cat violations evoked more activity than the ArgStr violation items for all the three prefixes, an effect that persisted across the entire time-window we analyzed.

#### Semantic Combination Analysis: 300–500 ms OribitoFrontal Cortex

From 300 to 500 ms, we performed another spatio-temporal 2 × 3 ANOVA between Prefix Type and Violation Type in the OF to investigate the semantic composition processing stage. When we conducted the analysis only on the trials that had been correctly rejected as impossible words of English, we identified separate clusters in left and right OF showing sensitivity to violation type, from 320 to 395 ms in the LH and from 355 to 400 ms in the RH. However, neither of these cluster tests survived correction for multiple comparisons (LH *p*_mcc_ = 0.2901, RH *p*_mcc_ = 0.1358).

Because as many as 25% of trials were incorrectly accepted in some conditions, we ran an additional spatio-temporal 2 × 3 ANOVA including all the trials. This analysis identified a LH cluster in inferior OF showing a prefix by violation.type effect from 325 to 440 ms (*p*_mcc_ = 0.056), such that for *out-* and *un-* items, Cat.Viol trials are more negative than ArgStr.Viol, but for *re-* the direction of the difference between conditions is reversed. The ANOVA results failed to survive correction for multiple comparisons (*p*_mcc_ = 0.114), however, the pairwise comparisons between the two violation types for the *out-* and *re-* items were robustly significant (*out-*: *t* = 3.55, *p*_mcc_ = 0.002; *re- t* = 2.21, *p*_mcc_ = 0.036), though the comparison for the *un-* items was not.

Table 7 reports the pairwise comparisons. Interestingly, as can be seen in Figure 2B, the difference between the two violation conditions goes in the opposite direction for *re-* and *out-* (with *un-* showing a weaker version of the *out-* pattern). While *re-* trials evoke the expected pattern based on the results from Greek (increased activity for the argument structure violation items than for the category violation items), the pattern is reversed for *out-* and *un-*, with more activation for the category violation items.

**TABLE 7:** Pairwise *t*-tests over time course in OF cluster area, and average value in cluster by condition.

	**Out-ArgStr.Viol**	**Re-Cat.Viol**	**Re-ArgStr.Viol**	**Un-Cat.Viol**	**Un-ArgStr.Viol**
Out-Cat.Viol	*t*_25_ = −3.55^∗^	*t*_25_ = −3.68^∗^	*t*_25_ = −1.62	*t*_25_ = −1.97	*t*_25_ = −2.52
	*p* = 0.002	*p* = 0.001	*p* = 0.117	*p* = 0.060	*p* = 0.019
Out-ArgStr.Viol		*t*_25_ = −0.27	*t*_25_ = 1.41	*t*_25_ = 0.86	*t*_25_ = 0.29
		*p* = 0.788	*p* = 0.172	*p* = 0.398	*p* = 0.774
Re-Cat.Viol			*t*_25_ = 2.21^∗^	*t*_25_ = 1.22	*t*_25_ = 0.82
			*p* = 0.036	*p* = 0.233	*p* = 0.422
Re-ArgStr.Viol				*t*_25_ = −0.36	*t*_25_ = −1.60
				*p* = 0.725	*p* = 0.123
Un-Cat.Viol					*t*_25_ = −0.73
					*p* = 0.470

*^∗^indicates significant tests after correction for multiple comparisons.*

## Discussion

Nearly 45 years after Taft and Forster (1975) first articulated the affix stripping model, we harnessed the combined spatial and temporal resolution of MEG to ask whether prefixed words were processed via the same mechanisms previous research identifies for suffixed words.

The answer turns out to be: mostly yes, but a little bit no. The profile revealed in the previous suffix processing studies (Fruchter and Marantz, 2015; Neophytou et al., 2018) distinguishes between (1) early, form-based decomposition, (2) lexeme lookup, (3) subcategorization-based grammatical licensing of affix+stem combinations, and (4) semantic well-formedness evaluation of the same combinations. Using the Neophytou et al. (2018) paradigm that allows us to investigate both decomposition and recomposition processes within the same experiment, we find the following:

### Decomposition Effects

#### Early, Visual Word Form-Based Sensitivity to Morpho-Orthographic Structure

Here we find that the stem|whole word transition probability measure, which consistently correlates with M170 activity for suffixed and pseudosuffixed words in the left hemisphere (Solomyak and Marantz, 2010; Lewis et al., 2011; Gwilliams and Marantz, 2018; Neophytou et al., 2018) correlates with evoked activity for prefixed words in the right hemisphere instead, and that the significant effect is about 50 ms later for the prefix experiment than the suffix experiment (Gwilliams and Marantz, 2018) run with the same subjects and within the same experimental sessions^5^. The lateralization effect may be related to the prefixes being initially routed to right primary visual areas, and the suffixes to the left, due to the distribution of the stimuli across the visual field (prefixes occur on the left, and are thus initially routed to the right visual hemifield, while right-adjoined suffixes are initially routed left). The only other MEG study to investigate the processing of prefixed words, Zweig and Pylkkänen (2009), finds that their suffixed words evoke a larger right lateralized M170 response than matched mono-morphemic control words, while their prefixed words evoke a larger response than undecomposable control words in both hemispheres. The relative timing delay in our experiment, and the lateralization differences between experiments both clearly merit further investigation. But it bears emphasizing that even a 210-ms effect of morphological complexity is an early effect, preceding later effects of stem frequency. Intriguingly, this temporal asymmetry is reminiscent of the results of Beauvillain (1996), who finds an earlier effect of cumulative root frequency on eye-movements for suffixes than prefixes.

The take away thus far seems to be that early, form-based morpho-orthographic analysis of prefixed words recruits neural resources in the right hemisphere, while such processing for suffixed words is more likely to be left-lateralized. But regardless of the lateralization, across both the Zweig and Pylkkänen (2009) studies, and the results from our participants (the current study and Gwilliams and Marantz, 2018), we find that the brain seems to be sensitive to the morphological constituents of both prefixed and suffixed stimuli at an early stage of processing. This early sensitivity is exactly what the Taft and Forster (1975) model would predict: both prefixed and suffixed words are processed via an early, visual word-form-based morphological decomposition process (affix stripping) that precedes subsequent constituent and whole word processing.

#### Lexeme Lookup Effects

We find an early stem frequency effect between 225 and 305 ms, in left inferior temporal cortex, but no early whole word frequency effect. This is consistent with Affix Stripping models in which decomposition precedes lexeme lookup, and only stems (not whole words) are stored in the lexicon.

### Recomposition Effects

#### Category-Based Licensing

We find a left hemisphere, posterior TL response sensitive to mismatches between the grammatical category required by the affix, and the category of the stem between 200 and 300 ms – the same time window and brain area associated with category-based licensing by Neophytou et al. (2018) for suffixed pseudowords in Greek. This region has also been associated with syntactic composition effects in sentence processing (Flick et al., 2018). There are no differences between the three prefixes we investigated in this study, despite the fact that the actual category of the “illegal” stems varied by prefix: while *re-* and *out-* were attached to unambiguous, monomorphemic adjectives to create category violating items, *un-* was attached to unambiguous, monomorphemic nouns. What appears to matter is simply whether the stem is the correct/expected category, though of course these results will need to be extended to affixes which select for other categories to confirm this conclusion.

#### Compositional Well-Formedness

We find a later, left hemisphere OF response sensitive to mismatches between semantic restrictions imposed by the affix and semantic properties of the stem, though these results are less statistically robust, and more variable than those found for Greek suffixes by Neophytou et al. (2018). The fact that the 300–500 ms OF semantic well-formedness response, which Neophytou et al. (2018) found to be greater for argument structure violation items than for category violation items for all three Greek suffixes in that study is less consistent across the prefixes in our study is interesting, but likely not related to the prefix vs. suffix distinction. We found that *re-* prefixed items evoked the same pattern as the Greek items, but that *out-* and *un-* evoked opposite direction results, with the category violation items triggering the larger responses. This unexpected result may be due to the fact that the *un-* and *out-* verbal prefixes are homophonous with other morphemes in the language. English also has a second *un-* prefix which attaches to adjectival bases (*unhappy*, uneventful), and the free-standing preposition *out* which can occur in lexicalized compounds (*outhouse*, outpost, outrage, outlaw). Its possible that the reason the category violating items prefixed with *un-* and *out-* are associated with larger OF responses than the argument structure violating items for those prefixes is that the existence of the competitor *un-* and *out* morphemes raises the possibility of an alternate analysis of those items. Participants may attempt to reanalyze *un-*_V_ as *un-*_A_ or *out-*_V_ as *out*_P_ in an attempt to resolve the mismatch between the category specifications of the prefix and stem. In a number of papers, Pylkkänen and colleagues find that OF activity is evoked by various kinds of grammatical coercion (Pylkkänen and McElree, 2007; Brennan and Pylkkänen, 2008, 2010; Pylkkänen et al., 2009a), so it may be the case that any argument structure violation response is swamped by a larger category coercion response for these two prefixes^6^. The fact that they robustly and quickly reject these items as possible words in the lexical decision task suggests that any such attempt at reanalysis fails to produce an acceptable whole word parse, so for now this possibility remains mere speculation. However, the fact that participants were slower to accept the grammatical items than the category violating items prefixed with *out-* and *un-* further suggests that there were late, decision-related difficulties with these two prefixes, not reflected in the earlier brain measures.

The other noteworthy result was the failure to find the expected pattern for response times and acceptance rates for the *re-* violation items. While all the other experiments using a similar affixation violation-type manipulation consistently find that grammatical category violations are rejected more robustly, and more quickly, than argument structure violations, in Greek (Manouilidou, 2007; Manouilidou and Stockall, 2014; Neophytou et al., 2018), English (Manouilidou and Stockall, 2014), and Slovenian (Manouilidou et al., 2016), no such effect is found for the *re-* prefixed items in the current experiment. This null effect is not a mere absence of a statistically significant effect attributable to noisy data (the SDs in the *re-* conditions were similar to those for the other prefixes), but the genuine absence of any hint of a difference between the two violation conditions in acceptance rate, and a trend to an opposite direction pattern in the response time. *Re-* argument structure violations were rejected more often and more quickly than the argument structure violating items prefixed with *un-* and *out-*. If we did not have the Greek results from Neophytou et al. (2018) for comparison, we might be tempted to relate this behavioral pattern to the OF activation patterns. In the 300–500 ms OF response, we find *out-* and *un-* items evoking the same pattern (Cat.Viol > ArgStr.Viol), while *re-* evokes an opposite direction pattern (ArgStr.Viol > Cat.Viol) – for each prefix, the violation type associated with the more robust OF response is also the violation type associated with the more robust (fast and consistent) behavioral response. This similar patterning might suggest that the OF response, which we have associated with morpho-semantic well-formedness, is a neural correlate of the lexical decision processes reflected in the behavioral responses. However, the contrast between the Greek and English results makes that unlikely. In Greek, across three different deverbal suffixes, and for the *re-* prefixed items in English, we see a clear association between syntactic category processing (or syntactic licensing) and TL and between OF and argument structure processing (or semantic recombination).

In this study, we tested a classic theory of linguistic processing by applying state-of-the-art analysis techniques to time-resolved neurophysiological data. Our goal in “re-revisiting” the question of whether prefixes are processed via the same mechanisms identified for suffixed words in other experiments was not merely to revisit the early, form-based affix-stripping question that has figured so prominently over the past 45 years, but also to advocate for a more multi-dimensional, wholistic and neurophysiologically grounded approach to the investigation of morphological processing. Models that are committed to affix stripping are also committed to a whole sequence of post-decomposition processes (at a minimum, the three identified here: lexeme lookup, category licensing, and semantic composition). While it is clear more research will be required to fully understand exactly how each of these processes works, the combined results of this study and the results of Neophytou et al. (2018) and Fruchter and Marantz (2015) confirm the viability of the approach. The similarity of the results across not only prefixes and suffixes, but also two different languages/grammatical systems, written with two different orthographies, is a significant finding, and arguably far more compelling than a similar processing profile for prefixes vs. suffixes within a language would be. A strong hypothesis can therefore be generated, and tested in future work: for all affixes, across all languages and all orthographic systems, we expect to find an early, fusiform gyrus sensitivity to stem:whole word transition probabilities (with the lateralisation of such an effect still requiring further investigation), a 200–400 ms temporal lobe sensitivity to stem frequency (but not to surface/whole-word frequency), and a 200–300 ms posterior temporal lobe sensitivity to violations of the affix’s grammatical category restriction. For verbal affixes, we further expect to find a 300–500 ms OF sensitivity to violations of the argument structure restrictions of the affix.

By comparing data collected in our experiment to a processing profile identified for suffixes in other experiments, our results provide credence to the hypothesis that prefix–stem combinations are processed through the same fundamental neural mechanisms previously identified for stem–suffix combinations, both at early (form-based) stages, and later (meaning-related) stages. This simple yet critical finding provides insight into the spatio-temporal dynamics of processing word structure, which appears to hold across different constructions as well as different languages.

## Data Availability

The datasets generated for this study are available on request to the corresponding author.

## Ethics Statement

This study was reviewed and approved by the NYU University’s Institutional Review Board (IRB) and carried out in accordance with its recommendations. All subjects gave written informed consent before beginning the experiment in accordance with the Declaration of Helsinki.

## Author Contributions

LS, CM, and AM conceived and designed the study. LS and LG selected and prepared the materials. LG wrote the script, collected the data, and made all figures. LS wrote and revised the majority of the manuscript. LS, LG, and KN processed and analyzed the data. KN and LG wrote sections of the manuscript. All authors contributed to the manuscript revision, and read and approved the submitted version.

## Conflict of Interest Statement

The authors declare that the research was conducted in the absence of any commercial or financial relationships that could be construed as a potential conflict of interest.

## Footnotes

1 Although see Steinhauer and Drury (2012) for an argument that the ELAN results may depend on specific design and signal analysis decisions which limit their generalizability.

2 www.neurobs.com

3 https://pythonhosted.org/eelbrain

4 Morphological affix frequency and orthographic affix/*n*-gram frequency are highly correlated, so could not be included in the same model. Rather one maximal model with the morphological variable, and one with the orthographic variable were run, and both were compared with a simpler model without this variable. No significant reduction in GoF was found for either comparison [*p*(χ^2^) > 0.4 in both cases], so neither variable was retained in the model.

5 It is worth emphasizing, however, that the Gwilliams and Marantz (2018) materials were not matched for any variables with ours, so there may be sources of variation other than the contrast between prefixation and suffixation behind the timing difference.

6 While the *re-* in such bound-stem, latinate words as receive, *remit*, reproach, etc. lacks the precise restitutive semantics and the main-stress-bearing properties of the *re-* in *refill* (to the extent we can say the bound, latinate *re-* has any semantics, it means something more like “backward” or “toward the subject”) it is nonetheless verbal, and therefore not category ambiguous in the way that *un-* and *out-* are. See Gwilliams and Marantz (2018) for a more detailed consideration of the processing of bound-stem items.
