# Supplementary material for: Prefix Stripping Re-Re-Revisited: MEG Investigations of Morphological Decomposition and Recomposition
Source: Front Psychol. 2019 Sep 6;10:1964. doi: 10.3389/fpsyg.2019.01964 (PMC6743348; doi:10.3389/fpsyg.2019.01964)
Supplement: Supplementary file 1 [file Table_1.DOCX]

Supplementary Material

Prefix Stripping Re-Re-Revisited: MEG investigations of Morphological Decomposition and Recomposition

Linnaea Stockall^*^, Christina Manouilidou, Laura Gwilliams, Kyriaki Neophytou, Alec Marantz

*** Correspondence:** Linnaea Stockall: l.stockall@qmul.ac.uk

# Supplementary Figures and Tables

## Materials: Critical Items

| Critical Items | | | | | | | | | | |
| --- | --- | --- | --- | --- | --- | --- | --- | --- | --- | --- |
| un- | | | | out- | | | re- | | | |
| grammatical | | cat.viol | sem.viol | grammatical | cat.viol | sem.viol | grammatical | cat.viol | sem.viol |  |
| 1 | unbalance | unaloe | unaccept | outact | outabrupt | outadvise | reacquire | reable | rebawl |  |
| 2 | unbend | unapple | unbathe | outargue | outabsurd | outamend | readjust | reacute | recheep |  |
| 3 | unblock | unaunt | unbicker | outbid | outajar | outarouse | reaffirm | readept | rechirp |  |
| 4 | unbolt | unbeach | unblow | outclass | outaloof | outbuck | realign | reample | rechortle |  |
| 5 | unbutton | unbeer | uncompete | outdance | outawkward | outcancel | reappear | reapt | recomplain |  |
| 6 | unchain | unbody | undiffer | outdare | outbad | outcause | rearrange | rearid | recroak |  |
| 7 | unclasp | uncabin | uneat | outdraw | outbizarre | outdodge | reassert | rebald | redie |  |
| 8 | uncloak | uncamel | unflatter | outdrink | outblue | outhave | reassign | rebleak | redoze |  |
| 9 | unclog | uncar | unflirt | outdrive | outbogus | outhold | reassure | rebrisk | reflounce |  |
| 10 | uncoil | unchasm | ungape | outearn | outcivic | outignore | reattach | rebrittle | refrown |  |
| 11 | uncork | uncider | ungawk | outfight | outcruel | outinform | rebuild | recasual | regiggle |  |
| 12 | uncover | uncoffee | ungovern | outfish | outdizzy | outkeep | recite | rechaste | regrumble |  |
| 13 | uncross | undebt | ungrow | outflank | outeerie | outkill | recollect | reclever | regurgle |  |
| 14 | uncurl | unduvet | unimagine | outfox | outentire | outlynch | recommit | recoarse | reholler |  |
| 15 | undo | uneagle | unjump | outgain | outfamous | outmake | recreate | recute | rehowl |  |
| 16 | undock | unfauna | unmelt | outgive | outfresh | outmuffle | redeem | redense | rehuff |  |
| 17 | undress | unfjord | unmove | outglow | outglad | outmurder | reelect | redevout | relaugh |  |
| 18 | unfasten | unfood | unnotice | outgross | outglum | outmuzzle | reenact | redual | repivot |  |
| 19 | unglue | unfoot | unoccupy | outlast | outhappy | outnourish | reenter | redumb | repray |  |
| 20 | unhitch | unfungi | unprepare | outmaneuver | outhoarse | outprovoke | refill | reeager | reshiver |  |
| 21 | unhook | ungnome | unprove | outnumber | outhot | outremind | refold | refeeble | resleep |  |
| 22 | unlace | ungoat | unshave | outpace | outirate | outshape | rehire | refickle | resniff |  |
| 23 | unlatch | ungrape | unshout | outperform | outlarge | outslay | reinstall | refierce | resnivel |  |
| 24 | unleash | unguest | unspeak | outrival | outlax | outsolve | reinvent | reforeign | resplurge |  |
| 25 | unload | unhoney | unspit | outshine | outlegal | outspot | relay | refraught | resputter |  |
| 26 | unlock | unhotel | unstop | outsmart | outmodern | outspurn | relearn | refrugal | resqueal |  |
| 27 | unpack | unhour | unswitch | outsource | outneat | outstifle | relive | regalore | retitter |  |
| 28 | unplug | unkite | untame | outspend | outpoor | outtell | relocate | regentle | retoddle |  |
| 29 | unroll | unlamp | unthank | outstare | outprim | outusurp | remarry | regenuine | rewhirr |  |
| 30 | unsaddle | unlimb | unthink | outstrip | outrich | oututter | resettle | rehonest | reyawn |  |
| 31 | unscrew | unmenu | untire | outweigh | outrude | outveto | resubmit | renimble | reyell |  |
| 32 | unseat | unpath | untrot |  |  |  |  |  |  |  |
| 33 | unstick | unpeat | untrust |  |  |  |  |  |  |  |
| 34 | unstitch | unpoem | unwear |  |  |  |  |  |  |  |
| 35 | untangle | unrabbit | unwork |  |  |  |  |  |  |  |

Table 1 Critical Items by Prefix and Condition

## Materials: Filler Items

| Fillers | | | | | | | | | | | | | | | | | | | | | |  |
| --- | --- | --- | --- | --- | --- | --- | --- | --- | --- | --- | --- | --- | --- | --- | --- | --- | --- | --- | --- | --- | --- | --- |
|  | re- | | | | | | | | | | un- | | | | | | | | | | |  |
|  | ungrammatical | grammatical | | | | | | | | ungrammatical | | | | grammatical | | | | | | | |  |
|  | cat.viol  substring | cat.viol  stem | verb.stem | | bound stem | | | cat.viol  substring | | | | cat.viol  stem | | | arg,struc.viol  stem | | | | verb.stem | | |  |
| 1 | reactual | reapologize | reacquired | | | rebuke | | | unatom | | | unatomic | | | | unboasting | | unbalancing | | | |  |
| 2 | realphabet | rebroaden | realigned | | | refute | | | unbaby | | | unbabyish | | | | uncheerful | | unblocked | | | |  |
| 3 | rebright | recriticize | reasserting | | | recruit | | | unbeast | | | unbeastly | | | | unclingy | | unbolted | | | |  |
| 4 | rebrutal | redeaden | reassigning | | | refrain | | | unbible | | | unbiblical | | | | uncomplaining | | unbucked | | | |  |
| 5 | recapital | redemonize | reattached | | | reprieve | | | unboy | | | unboyish | | | | uneatable | | unchained | | | |  |
| 6 | recentral | redramatize | recommitting | | | retrieve | | | undruid | | | undemonic | | | | unfinished | | unclasping | | | |  |
| 7 | recheap | refatten | redrawn | | | reconcile | | | unfable | | | undigital | | | | unfocused | | uncloaked | | | |  |
| 8 | recivil | refinalize | reelecting | | | replenish | | | unfaith | | | undramatic | | | | ungalloped | | uncoiling | | | |  |
| 9 | recritic | regladden | reenacted | | | repudiate | | | unfame | | | undruidic | | | | ungawking | | uncovering | | | |  |
| 10 | recrystal | rehumanize | reentered | | | retaliate | | | unfate | | | unfabled | | | | ungrinded | | uncrossed | | | |  |
| 11 | recustom | reidolize | regrown | | | resemble | | | unfibre | | | unfateful | | | | ungrumbling | | uncurled | | | |  |
| 12 | redeaf | reionize | rehired | | | remedy | | | unflesh | | | unfibrous | | | | unhelpful | | unfastened | | | |  |
| 13 | redigit | relegalize | reinstalled | | | rescue | | | unfruit | | | unflavorful | | | | unjoined | | unglueing | | | |  |
| 14 | reeconomy | relocalize | reinventing | | | reflect | | | ungirl | | | unfleshy | | | | unlovable | | unhitching | | | |  |
| 15 | reenergy | remagnetize | relearnt | | | rejoice | | | ungland | | | unfruity | | | | unmended | | unlacing | | | |  |
| 16 | refertile | rememorize | relived | | | regulate | | | unglass | | | ungirlish | | | | unpouting | | unlatching | | | |  |
| 17 | reformal | remodernize | remaking | | | receive | | | unglobe | | | unglandular | | | | unpraying | | unplugged | | | |  |
| 18 | reheight | reneaten | remelted | | | reckon | | | unhabit | | | unglassy | | | | unsneezing | | unscrambling | | | |  |
| 19 | reideal | repopularize | rereading | | | resume | | | unhair | | | unglobal | | | | unsniffed | | unsealing | | | |  |
| 20 | reitem | requicken | reselling | | | resist | | | unhealth | | | ungraceful | | | | unswimmable | | unseated | | | |  |
| 21 | relength | reripen | resending | | | retain | | | unhero | | | unhairy | | | | untilting | | unsheathed | | | |  |
| 22 | remagnet | resadden | resubmitted | | | reject | | | unidiom | | | unheroic | | | | unusable | | unsticking | | | |  |
| 23 | rememory | resocialize | retaken | | | revolve | | | unidiot | | | unidiotic | | | | unwailed | | unstitched | | | |  |
| 24 | repenal | resoften | retelling | | |  | | | unjaunt | | | unjaunty | | | | unwalked | | unthawed | | | |  |
| 25 | resick | resweeten | retrying | | |  | | | unlady | | | unjoyful | | | | unwandering | | untieing | | | |  |
| 26 | restable | revictimize | reusing | | |  | | | unlogic | | | unmealy | | | | unwashed | | untucked | | | |  |
| 27 | reterror | reweaken | rewashed | | |  | | | unmeal | | | unrobotic | | | | unwritten | | untwisting | | | |  |
| 28 | retough | reworsen | rewrapped | | |  | | | unmeat | | | unskillful | | | | unyelling | | unwrinkling | | | |  |
| 29 | revital |  |  | | |  | | | unmyth | | |  | | | |  | |  | | | |  |
| 30 | revocal |  |  | |  | | | unodor | | | |  | | |  | | | | |  | |  |
| 31 | rewide |  |  | |  | | | unsorrow | | | |  | | |  | | | | |  | |  |
| 32 |  |  |  | |  | | | unsuccess | | | |  | | |  | | | | |  | |  |
| 33 |  |  | |  | | |  | untact | | | | |  | | | |  | | | |  | |
| 34 |  |  | |  | | |  | unweek | | | | |  | | | |  | | | |  | |
| 35 |  |  | |  | | |  | unwife | | | | |  | | | |  | | | |  | |

Table 2 Filler Items. These items were designed for a separate experiment, and were not analysed for the experiment reported in this manuscript.

**
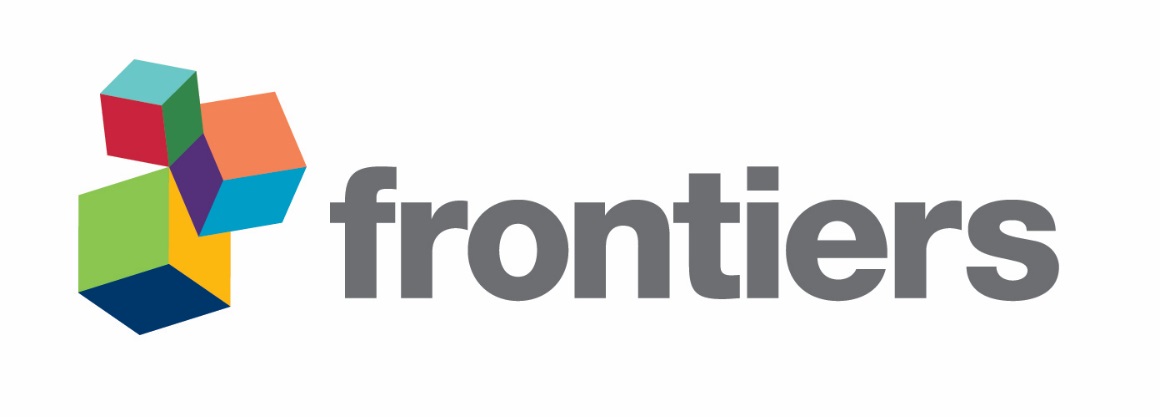
**
